# Determination of reference values and frequency of occurrence of patella alta in German shepherd dogs: a retrospective study

**DOI:** 10.1186/s13028-017-0304-1

**Published:** 2017-05-31

**Authors:** Anna Łojszczyk-Szczepaniak, Piotr Silmanowicz, Renata Komsta, Zbigniew Osiński

**Affiliations:** 1 Laboratory of Radiology and Ultrasonography, Department and Clinic of Animal Surgery, Faculty of Veterinary Medicine, University of Life Sciences in Lublin, Głęboka 30, 20-612 Lublin, Poland; 2 Department and Clinic of Animal Surgery, Faculty of Veterinary Medicine, University of Life Sciences in Lublin, Głęboka 30, 20-612 Lublin, Poland; 3 National Veterinary Research Institute in Pulawy, Partyzantów 57, 24-100 Puławy, Poland

**Keywords:** Patella alta, Vertical position of the patella, Insall Salvati, Patellar instability

## Abstract

**Background:**

Patella alta and patella baja are important conditions underlying a predisposition to many joint diseases, including patellar luxation and patellar chondromalacia of the articular cartilage. The frequencies of patella alta and patella baja have not yet been determined. The objectives of this study were to determine the frequency of patella alta and to determine reference values to the position of the vertical patella according to two modified techniques of the Insall–Salvati method in a group of 65 German shepherd dogs (115 stifle joints).

**Results:**

The upper limits of reference values for the normal vertical position of the patella were 1.79 and 2.13, depending on the method of measurement. A high prevalence of patella alta was observed in the group of German shepherd dogs. A correlation was demonstrated between the classification of dogs’ joints in the patella alta group and the multiplied risk of canine hip dysplasia (CHD) through the estimation of odds ratios.

**Conclusions:**

Dogs with patella alta were healthy dogs that did not exhibit orthopaedic problems in the stifle joints. The results revealed that the risk of CHD is twice as high in dogs with higher patellar ligament length to patella length ratio.

## Findings

The Insall–Salvati (IS) method is one of the most used diagnostic methods for identifying proximodistal malalignment of the patella in the femoral trochlea i.e. high-riding patella (patella alta [PA]) and low-riding patella (patella baja) [1–6]. In particular, the confirmation of a high vertical position in PA cases is clinically important as this is one of the conditions that may cause pain in the stifle joint and predispose to other joint diseases [6–8]. In PA, the alteration of the contact areas between the patella and the femoral trochlea leads to incongruence in the patellofemoral joint, increased patellofemoral stress during motion, chondromalacia of the articular cartilage and development of degenerative changes of the joint [2, 6–9]. It has been shown that a PA plays a role in development of medial dislocation of the patella, while a low-riding patella plays a role in lateral patellar dislocation, particularly in large dog breeds [2–5, 10–14]. Undiagnosed PA before surgical procedures for patella dislocation is also a major reason underlying the failure of surgery, which can reach 30–48% [5, 12, 13].

In dogs, the normal vertical position of the patella was first described in 2002 using the IS method [2]. Since then, several veterinary surgical studies have described the vertical position of the patella by employing different modified measurements, but the frequency of PA has not yet been established [1–5].

The aims of this study were to determine the frequency of PA in a population of German shepherd dogs and to obtain reference values for the vertical patella position in this breed.

A non-randomised observational study was performed among clinically normal police dogs. The recruited dogs belonged to the Lublin province and all German shepherd police dogs (58 males and 7 females) in this province were included. Animals weighted 22–44 kg (mean 35.8) and age ranged from 2 to 12 years (mean 5.05). All of the dogs participated in a university screening programme for orthopaedic diseases, including diseases of the hip, stifle and elbow joints. The screening programme included orthopaedic examination and radiological evaluation of these joints. Only radiographs of the stifle and hip joints were included in this study.

Approximately 20 min before radiological examination, each dog was sedated intramuscularly with 2 mg/kg xylazine, 0.25 mg/kg diazepam, and 0.05 mg/kg atropine sulphate. Mediolateral, bilateral stifle radiographs were taken with the stifle joint in a semi-flexed, unforced position. The ventrodorsal radiographs of the pelvis for hip joint evaluation were performed in accordance with the Fédération Cynologique Internationale (FCI) regulations (position 1) [15].

The radiographs were converted from analogue to a digital format using a scanner (ScanMaker 9800 XL Microtek TMA 1600) and analysed with imaging software (Iris Laboratorium Professional version, medi.com). Oblique stifle radiographs or radiographs with visible disease of the stifle joints were excluded from the study (15 stifle joints). The dog’s identity was not blinded on the radiographs.

**Fig. 1:**
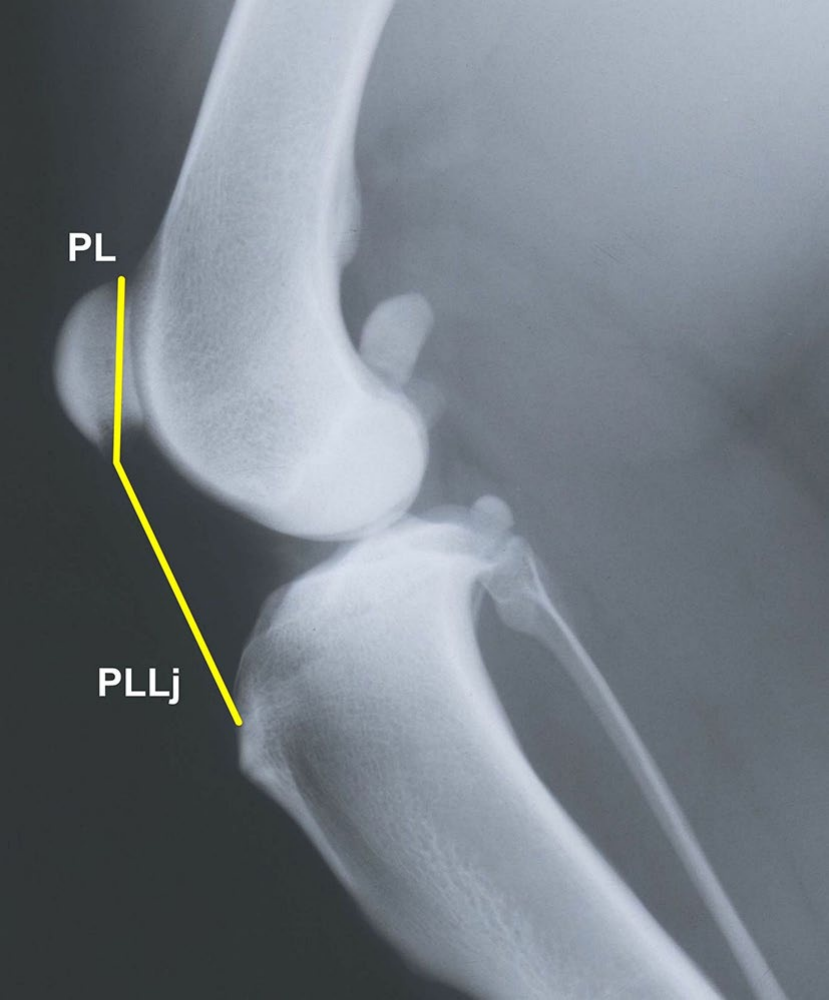
Medio-lateral radiograph of the stifle joint. The *lines* show measurements of the patellar length (PL) and patellar ligament length (PLLj) according to [2]. The measurements are used to calculate the Insall–Salvati ratio (PLLj/PL). The patellar ligament length is measured from the most distal part of the patella to the small indentation on the tibial tuberosity

**Fig. 2:**
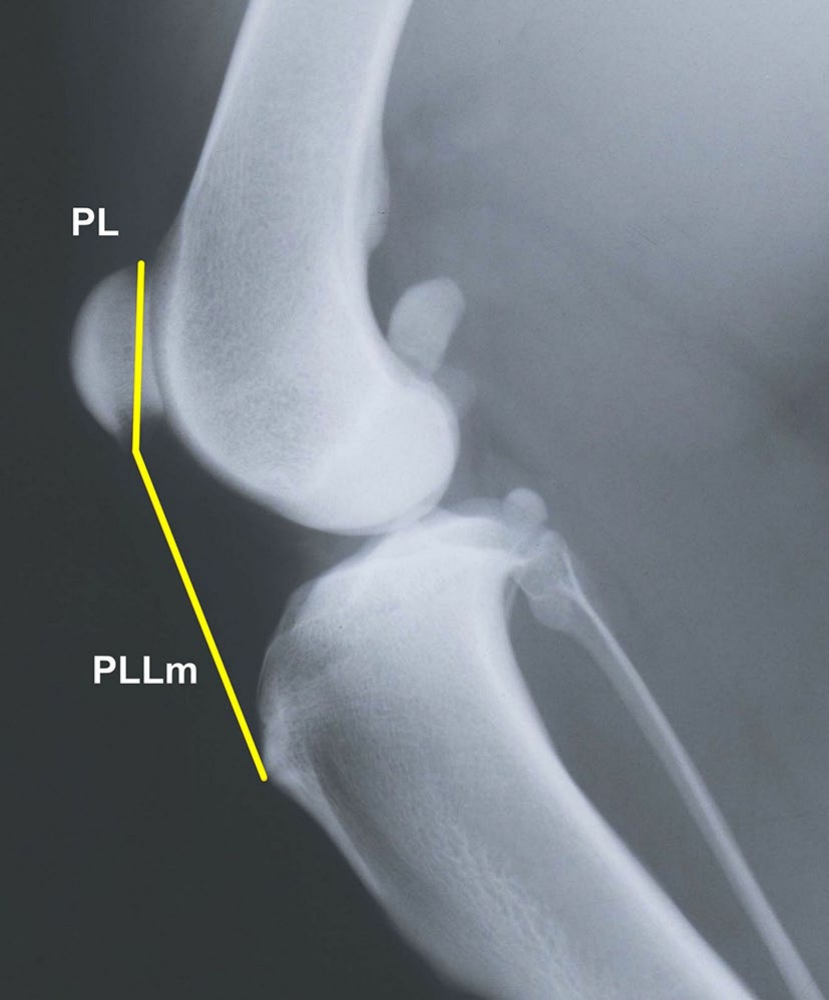
Medio-lateral radiograph of the stifle joint. The *lines* show measurements of the patellar length (PL) and patellar ligament length according to Mostafa et al. [5] (PLLm). The measurements are used to calculate the Insall–Salvati ratio (PLLm/PL). The patellar ligament length is measured from the most distal part of the patella to the proximal extent of the tibial tuberosity

The following measurements were obtained:

- The angle of flexion of the stifle joint based on the method described by Mostafa et al. [5]; and
- The vertical patellar position. Two methods for calculating the ratio of the patellar ligament length (PLL) measured in mm to patellar length (PL) in mm were employed: the method described by Johnson et al. [2] (PLLj/PL) (Fig. 1) and the modified method presented by Mostafa et al. [5] (PLLm/PL) (Fig. 2).

All measurements were made by one observer (ALS) and repeated two times. The mean values were used. The hip joint evaluation was performed by another observer (RK). Each hip joint was radiologically scored according to the FCI definition of canine hip dysplasia (CHD) (Table 1) [16].

**Table 1:** Canine hip dysplasia (CHD) score for each hip joint in examined dogs

CHD score according to FCI	Number of hip joints
A (no signs of hip dysplasia)	10
B (near normal hip joints)	34
C (mild hip dysplasia)	38
D (moderate hip dysplasia)	24
E (severe hip dysplasia)	9

*FCI* Fédération Cynologique Internationale

**Fig. 3:**
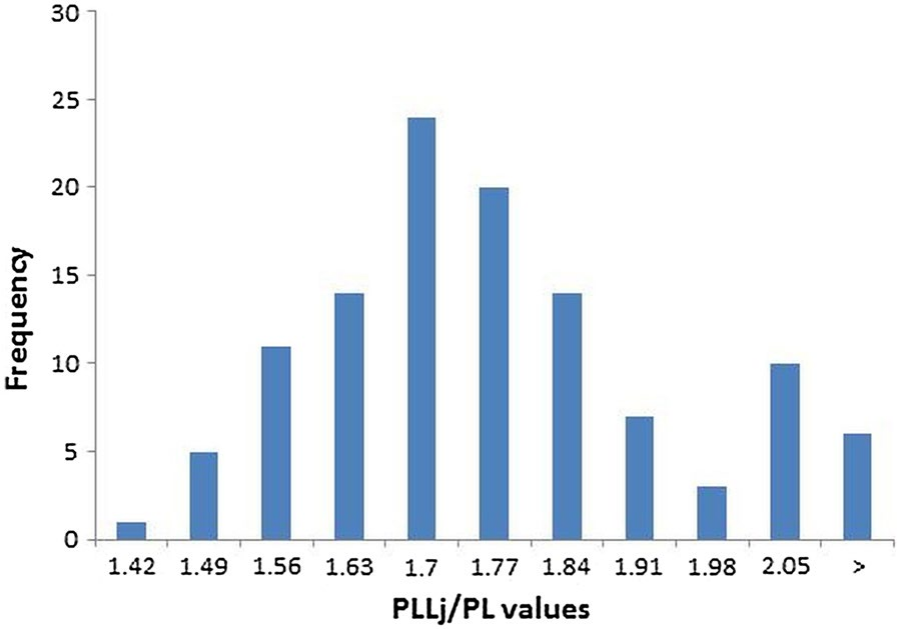
Histogram of PLLj/PL frequency. Number of dogs (frequency) with different PLLj/PL values. PLLj/PL—ratio of the patellar ligament length to the patella length according to [2]

**Fig. 4:**
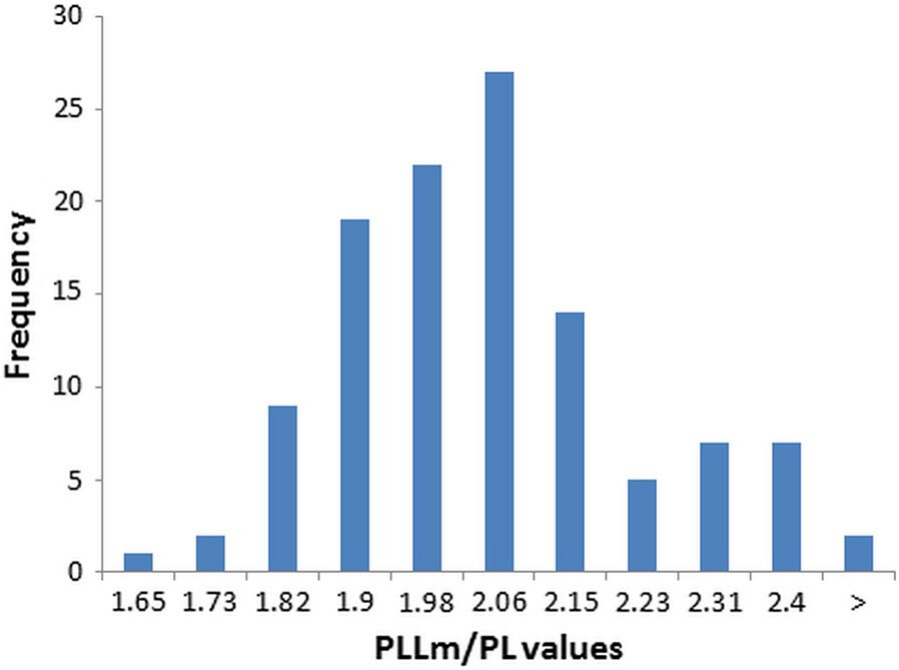
Histogram of PLLm/PL frequency. Number of dogs (frequency) with different PLLm/PL values. PLLm/PL—ratio of the patellar ligament length to the patella length according to [5]

The results of PLLj/PL and PLLm/PL calculations are presented in Figs. 3 and  4 as a function of their frequency in the joints of the studied dogs. In each histogram, two groups with either high or low PLL/PL values were recognised. These were a graphic combination of two distributions with the separation threshold between 1.91 and 1.98 for PLLj/PL and between 2.15 and 2.23 for PLLm/PL. In order to determine the division for the decreased and increased values, which characterise the studied joints, a cluster analysis with an EM Algorithm was applied. The analyses resulted in two normal distribution charts for each of the two methods for calculating PL, i.e. PLLj, and PLLm. Additionally, the statistical mean, standard deviation (SD), and minimum (Min) and maximum (Max) values were calculated for PL, PLLj and PLLm.

Reference values were determined as the double SD of PLLj/PL and PLLm/PL with a 95% confidence interval (CI). The usefulness of the reference values was evaluated for the normal patellar position and high vertical position of the patella groups (“normal” vs. “abnormal” groups) and for each of the two calculation methods [2, 5] by estimation of a one-dimension logistic regression with the odds ratio (OR) of the occurrence of CHD.

Statistical analysis and graphic illustrations were done using standard software programmes (Microsoft Excel 2010, Microsoft Corp.; Statistica 8.0, StatSoft Inc.).

The cluster analyses for calculations done with the Johnson’s method [2] and Mostafa’s method [5] are presented in Table 2. Twenty-three dogs (representing 38 stifle joints) had a high vertical position of the patella. With the exception of seven stifle joints, dogs exhibiting high PLLj/PL values possessed high PLLm/PL values as well. Thus, a confirmation by both ratios (PLLj/PL, PLLm/PL) indicated that 16 dogs (31 stifle joints) had a high patella position, corresponding to 27.0% of the examined dogs.

**Table 2:** Main results of the cluster analysis

	Normal group–normal patellar position	Abnormal group–high vertical patellar position
Johnson’s method	77 joints of mean value of PLLj/PL of 1.64	38 joints of mean PLLj/PL value of 1.93
Mostafa’s method	82 joints, mean PLLm/PL value of 1.93	33 joints, mean PLLm/PL value of 2.24

Obtained PLL/PL reference ranges with 95% CI		
Johnson’s method	1.46–1.79	1.72–2.1
Mostafa’s method	1.72–2.13	2.02–2.45

*PLL/PL* ratio of the patellar ligament length to the patella length

*PLLj/PL* ratio of the patellar ligament length to the patella length according Johnson et al. [2]

*PLLm/PL* ratio of the patellar ligament length to the patella length according Mostafa et al. [5]

*CI* confidence interval

There was no association between the degree of stifle joint flexion and PLLj/PL and PLLm/PL ratios, respectively. When applying the defined reference values for predictions of “normal” and “abnormal” groups, the OR values were 1.61 (0.64–4.06) and 2.11 (0.83–5.4) for Johnson’s and with Mostafa’s methods, respectively.

**Fig. 5:**
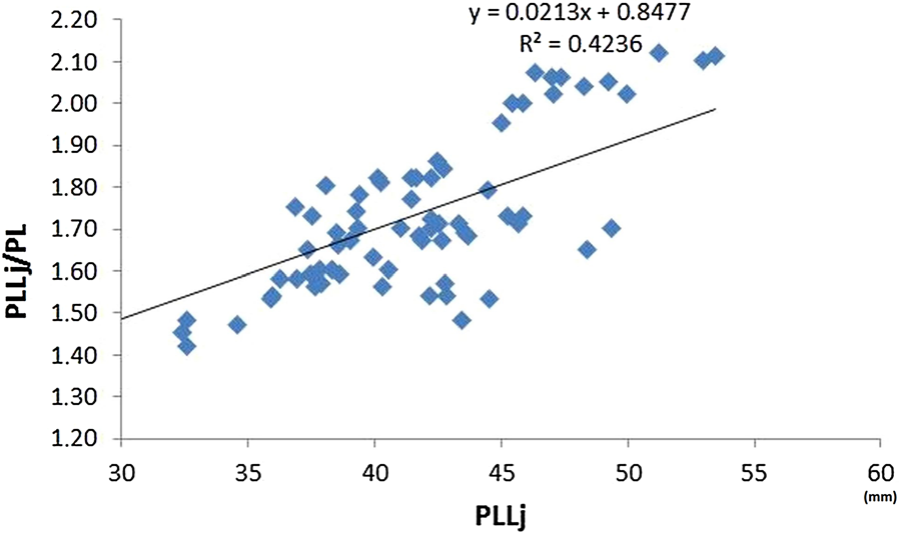
Relationship between the patellar ligament length (PLLj) and the patellar index (PLLj/PL). PLLj—patellar ligament measured in mm from the most distal part of the patella to the small indentation on the tibial tuberosity according to [2]. PLLj/PL*—*ratio of the patellar ligament length to the patella length according to [2]

**Fig. 6:**
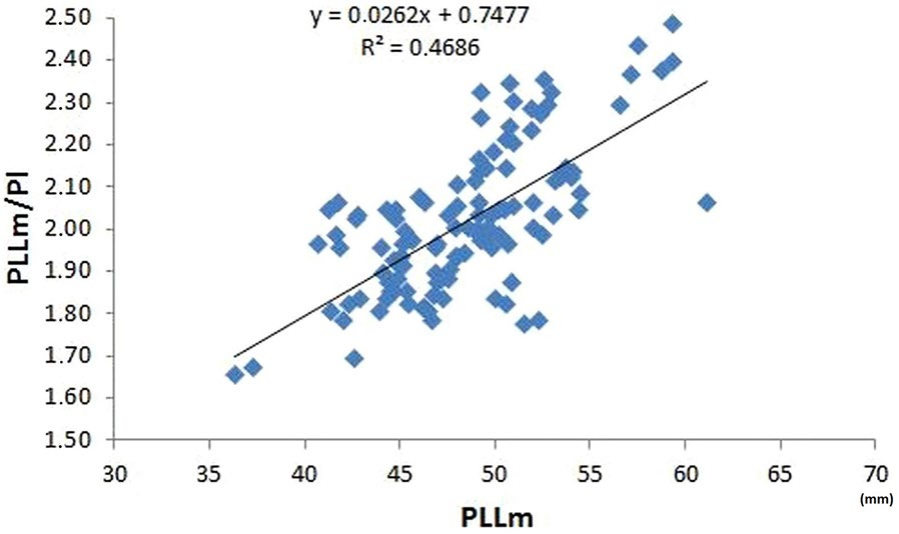
Relationship between the patellar ligament length (PLLm) and the patellar index (PLLm/PL). PLLm—patellar ligament length measured in mm from the most distal part of the patella to the proximal extent of the tibial tuberosity (according to Mostafa et al. [5]). PLLm/PL—ratio of the patellar ligament length to the patella length according Mostafa et al. [5]

We found a noticeable effect of an increased PLL on the patellar index, in contrast to the effect of the PL, characterised by lower variability (Figs. 5,  6). The ligament length of dogs with normally positioned patella and PA are shown in Table 3.

**Table 3:** Length of the patella and patellar ligament measured in mm in German shepherd dogs (n = 115)

	Mean in all animals	Standard deviation in all animals (±)
PLLm^a^	48.53	4.54
PLLj^b^	41.85	4.78
PL^c^	24.14	1.57

	Normal group			Patella alta group		
	Min	Max	Mean	Min	Max	Mean
PLLm^a^	36.32	61.16^d^	47.55	49.22	59.36	53.46
PLLj^b^	19.23	49.37	40.79	41.79	53.46	47.12

^a^Patellar ligament length measured from the most distal part of the patella to the proximal extent of the tibial tuberosity according to [5]

^b^Patellar ligament measured from the most distal part of the patella to the small indentation on the tibial tuberosity according to [2]

^c^Patellar length

^d^In the group of dogs with normal patellar positions, the maximum patellar ligament length was 54.49 mm (except for one dog with a patellar ligament length of 61.16)

**Fig. 7:**
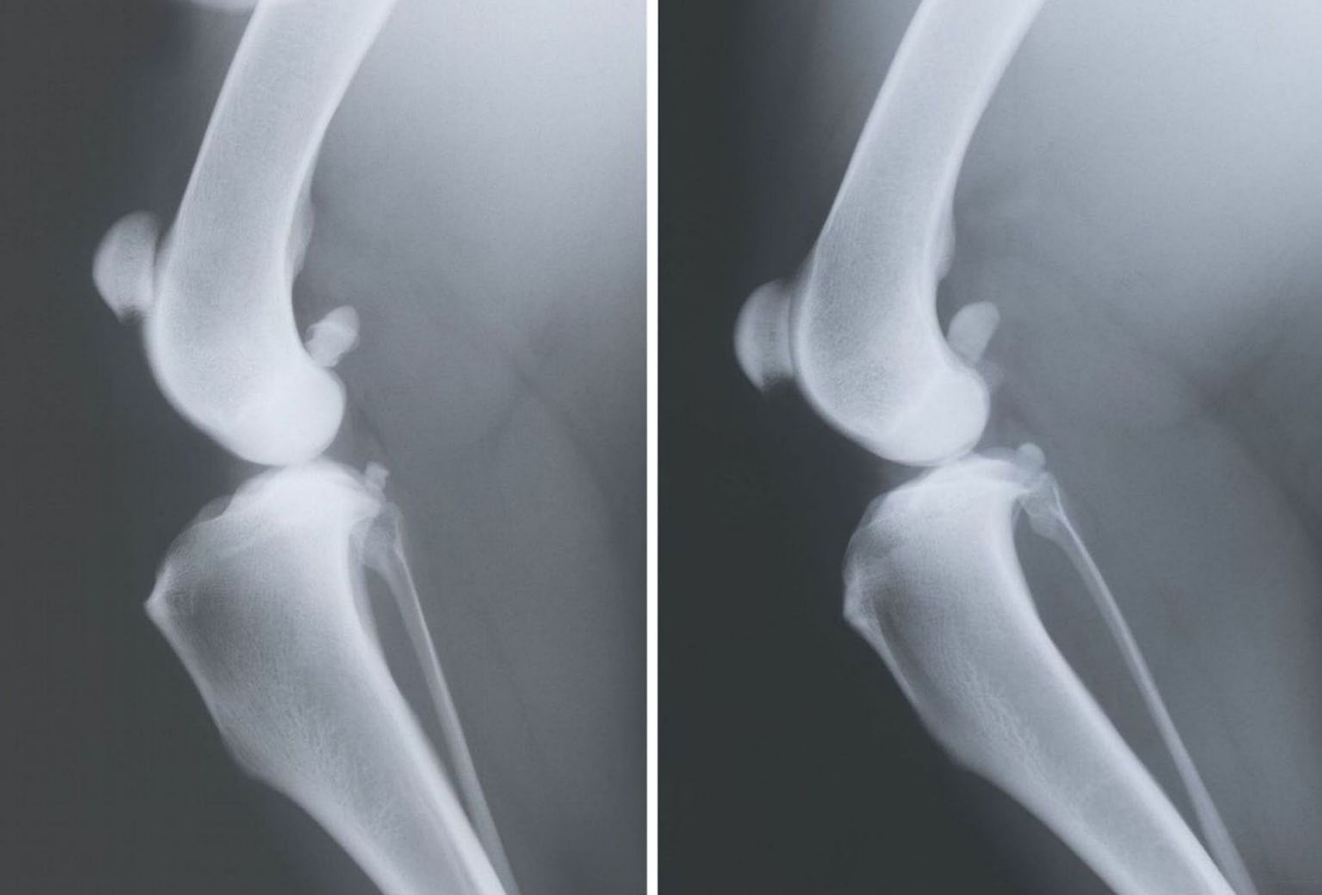
Medio-lateral radiographs of two dogs with different patellar position. The dog to the* right* has a normal patellar position and the dog to the *left* has a high vertical position of the patella (patella alta)

Using published reference values [1, 5], 19 dogs (34 stifle joints) had a high vertical position of the patella (Fig. 7). Of the 34 stifle joints, which exhibited PA according to [5], 17 were shown also to have PA by the method of Johnson et al. [1], (10 dogs, 15.4%).

The present study was so far performed in the largest and most homogeneous group of dogs. In other studies, a tendency towards higher index values in dogs with high body masses was observed, which may suggest that the breed, size and body shape influences the results [4]. Similar studies are therefore required for other breeds than German shepherd dogs.

In this study, the graphic analysis of the PLL/PL values (histograms) confirmed that it is possible to separate German shepherd dogs into groups. These groups may be defined as “normal” with low values of PLL/PL and “abnormal” with high values. A cluster analysis was applied for correct allocation and estimation of reference values for the two methods (Johnson vs. Mostafa). Thus, a correlation was proven between the classification of dogs’ joints in the “abnormal” group, with high patella position (PA), and the multiplied risk of CHD through estimating OR. The results showed more than double the risk of CHD in dogs characterised by higher PLL/PL indicators, estimated using the Mostafa method.

We also observed differences in the minimum and maximum values of the patellar ligament, which may indicate that a high patellar index may result from an excessively long patellar ligament as known for humans. The results indicate that the percentage of affected animals could be high, although the clinical significance of this condition should be studied in more detail. Given that the examined dogs with PA were clinically healthy dogs lacking clinical signs of lameness or pain in the stifle joint but with a higher occurrence of CHD, the significance of a PA should be further investigated.

Based on the reference values and published values, it was found that 15.4–27.0% of the German shepherd dogs had a high vertical position of the patella. The upper limit of the obtained PLLm/PL values was very similar to those proposed by Mostafa et al. [5], which supports that PA can be diagnosed in dogs based on a PLLm/PL ratio above 2.1. Compared with the values of Johnson et al. [1], the upper limit of the reference values differs considerably, whereas the lower limit is comparable. Such differences may be due to difficulties in assessing the position of the indentation on the tibial tuberosity. These difficulties may be due to the ligamentous notch not being located on the cranial edge of the tibial tuberosity but the medial side; therefore, strict medio-lateral radiographs do not always allow its visualisation [17]. The best method to calculate the height of the patella position is to use reference points, which are easy to identify. Using 2.1 for the patella position height as the upper limit for German shepherd dogs is justified.

## Abbreviations

CHD: canine hip dysplasia
CI: confidence interval
IS: Insall–Salvati Index, patellar index
Max: maximum values
Min: minimum values
OR: odds ratio
PA: patella alta
PL: patellar length
PLL: patellar ligament length
PLL/PL: ratio of the patellar ligament length to the patella length
PLLj/PL: ratio of the patellar ligament length to the patella length according Johnson et al. [2]
PLLm/PL: ratio of the patellar ligament length to the patella length according Mostafa et al. [5]
SD: standard deviation

## References

[1] Johnson AL, Broaddus KD, Hauptman JG, Marsh S, Monsere J, Sepulveda G (2006). Vertical patellar position in large-breed dogs with clinically normal stifles and large-breed dogs with medial patellar luxation. Vet Surg.

[2] Johnson AL, Probst CW, De Camp CE, Rosenstein DS, Hauptman JG, Kern TL (2002). Vertical position of the patella in the stifle joint of clinically normal large-breed dogs. Am J Vet Res.

[3] Kňayowický D, Ledecký V, Hluchý M, Ďurej M (2012). Use of modified Insall Salvati method for determination of vertical patellar position in dogs with and without cranial cruciate ligament rupture considering the morphology of the cranio-proximal tibia. Acta Vet Brno.

[4] Miles JE, Dickow M, Nielsen DH, Jensen BR, Kirpensteijn J, Svalastoga EL (2012). Five patellar proximodistal positioning indices compared in clinically normal 41 Greenland sled dogs. Vet J.

[5] Mostafa AA, Griffon DJ, Thomas MW, Constable PD (2008). Proximodistal alignment of the canine patella: radiographic evaluation and association with medial and lateral patellar luxation. Vet Surg.

[6] Pugliese LC, Pike FS, Aiken SW (2015). Distal tibial tuberosity translation using TTA implants for the treatment of patella alta in large breed dogs. Vet Comp Orthop Traumatol.

[7] Barnett AJ, Prentice M, Mandalia V, Wakeley CJ, Eldrige JD (2009). Patellar height measurement in trochlear dysplasia. Knee Surg Sports Traumatol Arthrosc.

[8] Kannus PA (1992). Long patellar tendon. Radiographic sign of patellofemoral pain syndrome—a prospective study. Radiology.

[9] Colvin AC, West RV (2008). Patellar instability. J Bone Joint Surg Am.

[10] Towle HA, Griffon DJ, Thomas MW, Siegel AM, Dunning D, Johnson A (2005). Pre- and postoperative radiographic and computed tomographic evaluation of dogs with medial patellar luxation. Vet Surg.

[11] Bound N, Zakai D, Butterworth SJ, Pead M (2009). The prevalence of canine patellar luxation in three centres. Vet Comp Orthop Traumatol.

[12] Edwards GA, Jackson AH (2012). Use of a TT plate for correction of severe patella baja in Chihuahua. J Am Anim Hosp Assoc.

[13] Gibbons SE, Macias C, Tonzing MA, Pinchbeck GL, McKee W (2006). Patellar luxation in 70 large breed dogs. J Small Anim Pract.

[14] Wangdee C, Torwattanachai P (2010). Lateral patellar luxation in three Pomerian dogs: a case report. Thai J Vet Med.

[15] Radiographic procedure for hip dysplasia evaluation. http://www.fci.be/medias/SCI-REG-DYS-HAN-PRO-en-619.pdf. Accessed 11 Jan 2017.

[16] Flückinger M (2007). Scoring radiographs for canine hip dysplasia—the big three organisations in the world. Eur J Companion Anim Pract.

[17] Lutnicki W (2005). Zarys osteologii zwierząt domowych (Compendium of Domestic Animals Osteology).

